# Broadening the spectrum of *NTRK* rearranged mesenchymal tumors and usefulness of pan-TRK immunohistochemistry for identification of *NTRK* fusions

**DOI:** 10.1038/s41379-020-00657-x

**Published:** 2020-08-28

**Authors:** Iva Brčić, Theresa Maria Godschachner, Marko Bergovec, Jasminka Igrec, Holger Till, Herwig Lackner, Susanne Scheipl, Karl Kashofer, Thomas Brodowicz, Andreas Leithner, Joanna Szkandera, Bernadette Liegl-Atzwanger

**Affiliations:** 1 Diagnostic and Research Institute of Pathology, Comprehensive Cancer Centre Graz, Medical University of Graz, Graz, Austria; 2 Department of Orthopedics and Trauma, Comprehensive Cancer Centre Graz, Medical University of Graz, Graz, Austria; 3 Division of General Radiology, Department of Radiology, Comprehensive Cancer Centre Graz, Medical University of Graz, Graz, Austria; 4 Department of Pediatric and Adolescent Surgery, Comprehensive Cancer Centre Graz, Medical University of Graz, Graz, Austria; 5 Division of Pediatric Hematology and Oncology, Department of Pediatrics and Adolescent Medicine, Comprehensive Cancer Centre Graz, Medical University of Graz, Graz, Austria; 6 Clinical Division of Oncology, Department of Medicine I, Comprehensive Cancer Centre, Medical University Vienna, Vienna, Austria; 7 Clinical Division of Medical Oncology, Department of Medicine, Comprehensive Cancer Centre Graz, Medical University of Graz, Graz, Austria

**Keywords:** Cancer genetics, RNA sequencing

## Abstract

Fusions involving *NTRK1*, *NTRK2*, and *NTRK3* are oncogenic drivers occurring in a spectrum of mesenchymal neoplasms ranging from benign to highly malignant tumors. To gain further insights into the staining profile with the pan-TRK assay, we analyzed a large number of soft tissue sarcomas and correlated our findings with molecular testing. Additionally, we expand the spectrum of *NTRK*-fusion tumors by reporting a mesenchymal lesion in the lung as well as a mesenchymal skin lesion in the spectrum of benign fibrous histiocytoma with *NTRK*—fusion. We retrospectively reviewed soft tissue sarcomas diagnosed at the Diagnostic and Research Institute of Pathology, Medical University of Graz, between 1999 and 2019, and cases from the consultation files of one of the authors (BLA). In total, 494 cases were analyzed immunohistochemically with pan-TRK antibody (clone EPR17341, RTU, Roche/Ventana) and positive cases (defined as any cytoplasmic/nuclear staining in more than 1% of tumor cells) underwent next-generation sequencing (NGS). Immunohistochemical staining was observed in 16 (3.2%) cases. Eleven cases with focal weak and moderate cytoplasmic/membranous or focal moderate to strong nuclear staining did not harbor an *NTRK*-fusion (three synovial sarcomas, three leiomyosarcomas, two extraskeletal myxoid chondrosarcomas, and one each: dedifferentiated liposarcoma, pleomorphic liposarcoma, and myxofibrosarcoma). Four cases showed strong diffuse nuclear and/or cytoplasmatic staining, and one case showed diffuse, but weak cytoplasmic staining. All these cases demonstrated an *NTRK*-fusion (*LMNA-NTRK1, IRF2BP2-NTRK1, TMB3-NTRK1, ETV6-NTRK3, RBPMS-NTRK3*). Pan-TRK assay (clone EPR17341, RTU, Roche, Ventana) immunohistochemistry serves as a reliable diagnostic marker that can also be expressed in non-*NTRK*-rearranged mesenchymal neoplasms. It can be used as a surrogate marker for identification of *NTRK* fusion, nevertheless, an RNA-based NGS for detection of the specific fusion should be performed to confirm the rearrangement, if patients are undergoing targeted therapy. Additionally, we identified *NTRK*-fusion-positive, primary mesenchymal tumors of the lung and the skin.

## Introduction

The tropomyosin-related/receptor kinase (Trk) family includes three transmembrane neurotrophin receptors TrkA (NTRK1), TrkB (NTRK2) and TrkC (NTRK3). They are encoded by *NTRK* genes (*NTRK1*, *NTRK2*, and *NTRK3*) located on human chromosomes 1q23.1, 9q21.33, and 15q25.3, respectively [1]. Oncogenic gene fusions involving *NTRK1-3* genes lead to a constitutive activation or overexpression of Trk receptors [2, 3]. The presence of *NTRK1-3* fusions has been identified as an agnostic marker for treatment response with a selective small-molecule inhibitor of the TRK kinases (TKI) in solid tumors. The *ETV6*-*NTRK3* gene fusion has been described in sporadic solid tumors, including congenital infantile fibrosarcoma (IFS), congenital “cellular” mesoblastic nephroma, secretory breast carcinoma, and carcinoma of the salivary gland (mammary analog secretory carcinoma) in more than 90% of cases and fusion detection has been used as diagnostic confirmation [4–11]. Treating these patients with small TKI demonstrated tremendous treatment responses [12–18].

Over the last years, new tumor entities, especially in the already very heterogenous group of soft tissue tumors, were described harboring *NTRK* fusions [6, 8, 19–21]. In addition, *NTRK* fusions were detected in other more common cancer types, such as papillary thyroid carcinoma, gastrointestinal stromal tumor (GIST), gliomas, non-small cell lung cancer (NSCLC), colorectal carcinoma (most frequently found in MSI-high carcinoma associated with *MLH1* promoter hypermethylation), as well as malignant melanomas, uterine sarcomas, and pancreatic adenocarcinomas, however in less than 1% of all solid tumors overall [3, 6, 22–35].

Targeted therapy with a kinase inhibitor leads to a response in a majority of patients with *NTRK1/2/3* gene fusion-positive cancers [12, 13, 18, 36] and because limited treatment options are available for the vast majority of soft tissue sarcomas detection of tumors harboring *NTRK1-3* fusions results in a tremendous treatment benefit. Therefore, it is necessary to find reliable and cost-effective techniques to select patients that likely will respond to these expensive treatment options. At the moment, RNA-based NGS screening approaches are the most reliable method to detect *NTRK1-3* fusions also with unknown partners. However, this technique is not universally available. Other screening methods for the detection of *NTRK*-fusion include immunohistochemistry (IHC) and different molecular assays (fluorescence in situ hybridization (FISH), reverse transcription-polymerase chain reaction (RT-PCR), combined DNA- and RNA-based next-generation sequencing (NGS)).

IHC is the least expensive and widely available method that can be used as a screening tool to search for TRK fusion protein expression in all tumor types. However, standardized interpretation scores are not available for pan-TRK IHC [29, 37–39]. IHC has its limitations by detecting the expression of the pan-TRK fusion protein as well as wild type protein, and the staining pattern is variable in intensity and localization of the identified pan-TRK protein [29, 38, 39]. Studies reported so far used different dilutions (1:300, 1:500) of the clone EPR17341 from Abcam, an antibody that needs to be evaluated individually in each laboratory.

All available antibodies detect the wild-type protein that has been demonstrated in smooth muscle, testis, and neural tissue resulting in a positive IHC expression, especially in a subset of tumors with neuronal and smooth muscle differentiation without identifiable fusion [29, 33, 39, 40]. Therefore, positive IHC results need to be evaluated by an RNA-based NGS method to confirm or exclude an *NTRK1/2/3* fusion. To get further insights into the staining profile with the pan-TRK rabbit monoclonal antibody (clone EPR17341, RTU, Assay, Roche/Ventana), we performed analysis on a large number of soft tissue sarcomas and correlated our IHC results with molecular testing.

## Material and methods

### Patients and tumor characteristics

We retrospectively analyzed 487 soft tissue sarcomas diagnosed at the Diagnostic and Research Institute of Pathology, Medical University of Graz, from 1999 until 2019. In addition, selected consultation cases of one of the authors (BLA) diagnosed from 2017 until 2019 were reviewed. Hematoxylin and eosin (H&E)-stained whole-tissue sections of all cases were reviewed by two soft tissue pathologists (IB and BLA) to confirm the diagnosis and select appropriate, viable tumor tissue areas for tissue microarrays (TMA) construction. Immunohistochemistry of all cases were reviewed by three pathologists (IB, TMG, and BLA). Institutional ethical approval was obtained from the Institution Review Board. Majority of samples used for this project have been provided by Biobank, Graz, Austria

Specimens included in the study comprised whole tumor sections of five consultation cases (all *NTRK*-rearranged sarcomas) and TMAs conducted at the Diagnostic and Research Institute of Pathology including a collection of various sarcoma types, including (total *n* = 489): myxofibrosarcomas (*n* = 116), soft tissue and uterine leiomyosarcomas (*n* = 82), undifferentiated pleomorphic sarcomas (UPS) (*n* = 55), synovial sarcomas (*n* = 45), myxoid liposarcomas (*n* = 37), atypical lipomatous tumors/well-differentiated liposarcomas (*n* = 34), dedifferentiated liposarcomas (*n* = 34), dermatofibrosarcoma protuberans (*n* = 19), low-grade fibromyxoid sarcomas (LGFMS) (*n* = 12), angiosarcomas (*n* = 10), SFT (*n* = 8), spindle cell sarcomas (*n* = 6), myxoinflammatory fibroblastic sarcoma (*n* = 5), pleomorphic liposarcoma (*n* = 4), alveolar soft part sarcomas (*n* = 4), spindle cell/pleomorphic sarcoma (*n* = 3), clear cell sarcoma (*n* = 3), extraskeletal myxoid chondrosarcomas (*n* = 2), embryonal rhabdomyosarcoma (*n* = 2), sclerosing epithelioid fibrosarcoma (*n* = 2), epithelioid sarcoma (*n* = 1), mesenchymal chondrosarcoma (*n* = 1), malignant peripheral nerve sheath tumor (MPNST) (*n* = 1), pleomorphic rhabdomyosarcoma (*n* = 1), malignant melanotic schwannoma (*n* = 1), and myoepithelioma (*n* = 1).

In addition, we re-analyzed the collective of spindle cell sarcomas (*n* = 6) and UPS (*n* = 55) in relation to the patients age, morphologic patterns and IHC profiles described in *NTRK*-fusion tumors. All HE stained slides and IHC were re-reviewed by two soft tissue pathologists (IB and BLA).

### Tissue microarray construction

TMA was constructed using a manual tissue microarray apparatus (Beechers Instruments, Sun Prairie, WI, USA). Four 0.6 mm cores were taken from each of the collected formalin-fixed, paraffin-embedded (FFPE) samples and embedded in a paraffin block. TMAs were constructed in duplicates. H&E-stained, 4 μm TMA sections were used to verify all samples.

### Immunohistochemical and molecular analysis

FFPE TMA blocks were cut in 4 µm-thick sections and placed on slides. IHC staining for pan-TRK expression was performed on the Benchmark Ultra platform (Ventana Medical Systems, Tucson, AZ) with iVIEW DAB Detection Kit (Ventana Medical Systems, Tucson, AZ), using a commercially available pan-TRK assay (rabbit monoclonal antibody, clone EPR17341, Assay, RTU, Roche, Ventana). Normal appendix and brain tissues were used as positive controls. The immunohistochemical evaluation included (i) location (membranous, cytoplasmic, nuclear, mixed), (ii) intensity (strong, moderate, weak, and negative) and (iii) the extent (percentage of positive tumor cells) of the IHC staining. Membranous, cytoplasmic, and nuclear staining patterns were considered positive if ≥1% of tumor cells exhibited positivity at any intensity above background [29]. Diffuse staining was defined as moderate to strong expression in ≥50% of tumor cells [39].

### RNA workflow

IHC positive cases with available FFPE material were sent for RNA-based analysis with Archer FusionPlex Sarcoma Panel to assess specific *NTRK1*, *NTRK2*, and *NTRK3* rearrangements for the production of NTRK fusion transcripts. For each sample, 5–8 × 10 μm FFPE sections were cut from a representative block, and macro dissection was performed with a scalpel to enrich for tumor content. According to the manufacturer’s instructions, RNA was isolated using the Maxwell RSC RNA FFPE kit.

### Targeted next-generation sequencing

RNA was quantified by ribogreen fluorescence, and 250 ng total RNA was used for the Archer Fusionplex Sarcoma Kit. NGS libraries were sequenced on Ion Torrent Proton using the Ion PI Hi-Q Sequencing 200 kit (Thermo Fischer, Waltham, MA). The analysis was performed with ArcherDX Analysis software Version 5.1.3.

## Results

### Tumors with immunohistochemical positive pan-TRK staining

**Fig. 1:**
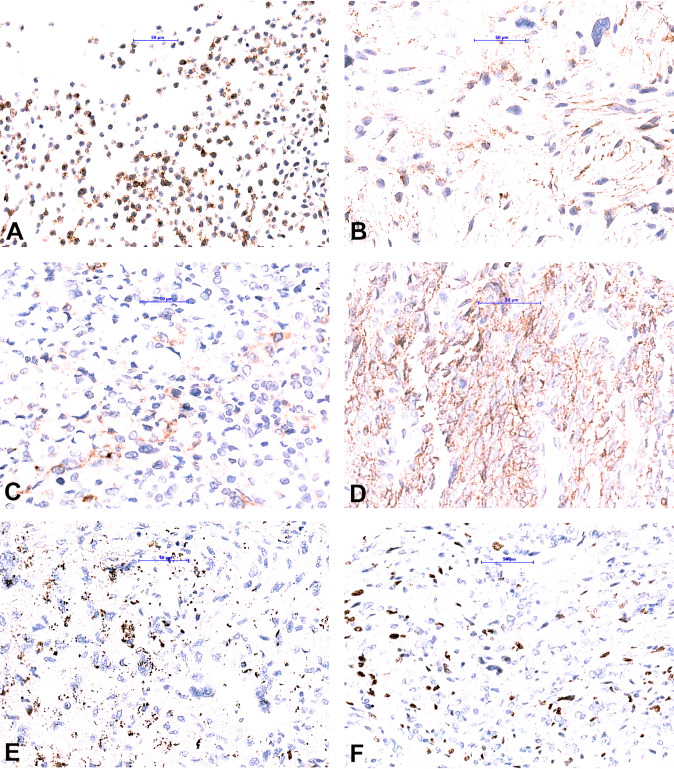
Pan-TRK expression patterns in non-*NTRK* fusion sarcomas. Focal weak cytoplasmic/membranous expression in the extraskeletal myxoid chondrosarcoma (**a**), myxofibrosarcoma (**b**), pleomorphic liposarcoma (**c**) and synovial sarcoma (**d**). Focal, moderate to strong pan-TRK expression with dot-like aggregates (**e**) and nuclear (**f**) staining in two different leiomyosarcomas.

From 494 tumors analyzed, 16 (3.2%) tumors stained positive, including 5 tumors with diffuse staining (further discussed in detail in the next paragraph) and 11 tumors with focal (in <50% of tumor cells) cytoplasmic/membranous or nuclear pan-TRK staining. The latter included 3/45 (6.7%) synovial sarcomas, 3/82 (6.7%) leiomyosarcoma (one uterine), 2/2 (100%) extraskeletal myxoid chondrosarcomas, 1/34 (2.9%) dedifferentiated liposarcoma, 1/4 (25%) pleomorphic liposarcoma, and 1/116 (0.9%) myxofibrosarcomas (Fig. 1). Patterns of staining included: (i) weak cytoplasmic/membranous expression in the extraskeletal myxoid chondrosarcoma, myxofibrosarcoma, dedifferentiated liposarcoma, pleomorphic liposarcoma, and synovial sarcoma; (ii) moderate to strong staining with dot-like aggregates or nuclear staining in leiomyosarcomas. Additionally, NGS was performed, and none of these 11 tumors harbored an *NTRK* fusion.

### UPS and spindle cell sarcoma subgroup

Within this patients collective of 55 UPS and 6 spindle cell sarcomas, the mean patients’ age was 70 years. Only 4 patients were under the age of 55 (the youngest patient was 50 years old). Among UPSs, only 8 cases showed partly a spindle cell morphology (in the age range 65–70 years). By immunohistochemstry, none of these cases demonstrated CD34 and S100 expression by IHC. In addition, pan-TRK IHC on whole-tissue slides was performed on all 16 cases and were negative, confirming the TMA results. Taking into account that some very rare cases of *NTRK*-fusion tumors were described to lack pan-TRK positivity by IHC, we additionally performed RNA-sequencing on all 4 patients under the age of 55, as the *NTRK*-fusion tumors most frequently occur in the younger age group. None of the cases showed a *NTRK*-fusion.

### Soft tissue tumors with diffuse pan-TRK protein expression by IHC and NTRK1-3 fusions by NGS

#### Clinical findings

The *NTRK*-rearranged sarcomas included five cases: one IFS, one lipofibromatosis-like neural tumor (LFLNT), one low-grade spindle cell sarcoma, one fibrohistiocytic proliferation of the skin (with unusual morphology for a benign fibrous histiocytoma) and one high-grade spindle cell sarcoma. The clinicopathological data are summarized in Table 1. In short, three patients were female (60%), and two patient was male (40%), with a median age of 20 years (age range 8 months to 48 years). Two tumors were located in the lower leg, and the remaining three were found in the forearm, lung, and skin (dermis).

**Table 1:** Summary of clinical, immunohistochemical, and molecular data.

Pts	Age	Sex	Diagnosis	Location	Fusion	Immunohistochemical staining	
						Intensity	Pattern
1	8 mo	f	Infantile fibrosarcoma	Forearm	*ETV6-NTRK3*	Strong	Nuclear
2	50 years	m	Lipofibromatosis-like neural tumor	Neck	*LMNA-NTRK1*	Strong	Cytoplasmic
3	15 years	m	Low-grade spindle cell sarcoma	Lung	*RBPMS - NTRK3*	Weak	Cytoplasmic
4	48 years	f	Fibrohistyocitic proliferation	Lower leg (dermis)	*IRF2BP2 -NTRK1*	Strong	Cytoplasmic
5	26 years	f	High-grade spindle cell sarcoma	Lower leg	*TMB3-NTRK1*	Strong	Cytoplasmic

*f* female, *m* male, *mo* months.

#### Morphological, immunohistochemical and molecular findings

The morphologic spectrum of the *NTRK* fusion tumors was heterogeneous.

1. IFS was characterized by monomorphic spindled to ovoid cells with small hyperchromatic nuclei arranged in sheets and intersecting fascicles with high mitotic activity (Fig. 2a–c). Immunohistochemical stains for SMA, desmin, CD34, ERG, S100, SOX10, EMA, pan-Keratin were negative. Pan-TRK showed diffuse strong nuclear and cytoplasmic staining in all tumor cells. The diagnosis of IFS was confirmed by the presence of a typical *ETV6-NTRK3* fusion.

**Fig. 2:**
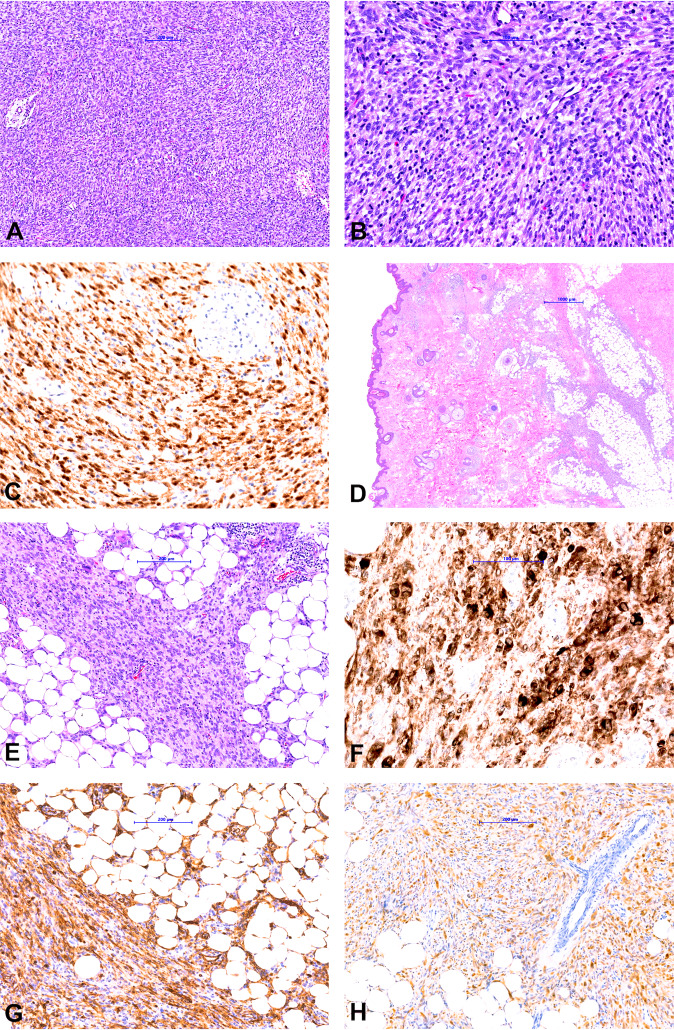
Morphological and immunohistochemical findings in cases 1 and 2. **a**, **b** Infantile fibrosarcoma composed of spindle cells with small hyperchromatic nuclei arranged in sheets and intersecting fascicles. **c** Pan-TRK staining shows diffuse strong nuclear and cytoplasmic staining in the tumor cells. **d**, **e** Lipofibromatosis-like neural tumor composed of spindle cells with mild nuclear atypia and pale eosinophilic cytoplasm arranged in streaming fascicles infiltrating the adipose tissue. **f** Pan-TRK staining shows diffuse strong cytoplasmic staining. Tumor cells show diffuse co-expression of CD34 (**g**) and S100 (**h**).
2. LFLNT was a subcutaneous lesion on the neck composed of spindle cells with mild nuclear atypia and pale eosinophilic cytoplasm arranged in streaming fascicles infiltrating the adjacent adipose tissue (Fig. 2d–h). Mitotic activity was low. The tumor showed co-expression of S100 and CD34. With the pan-TRK antibody, a strong diffuse cytoplasmic staining pattern was observed. NGS revealed an *LMNA-NTRK1* fusion (Fig. 3).

**Fig. 3:**
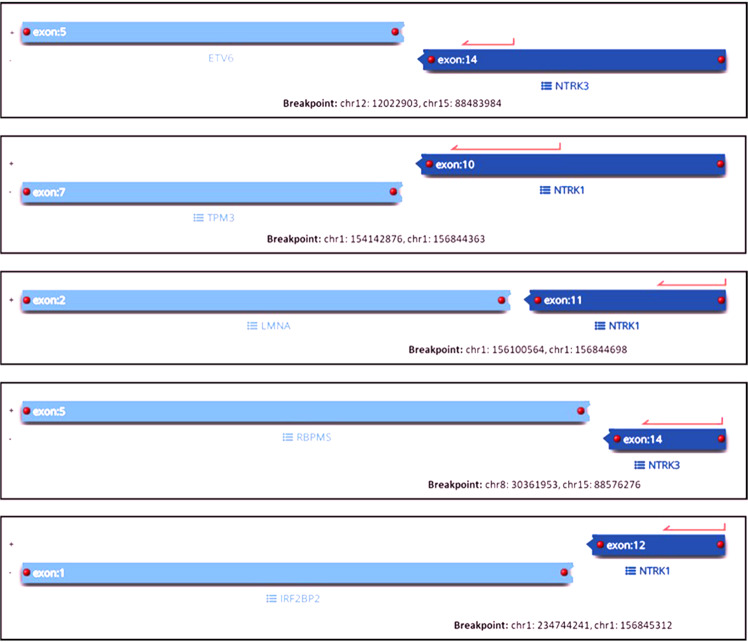
Visualization of the different NTRK fusions analyzed by NGS. Images show the genes and exons involved in the translocation event as well as the genomic location of each of the five different *NTRK* fusion.
3. Skin fibrohistiocytic tumor was a dermal lesion composed of epithelioid and spindle tumor cells resembling an unusual benign fibrous histiocytoma like lesion composed of cells with mild nuclear atypia and eosinophilic cytoplasm with low mitotic activity (Fig. 4a–c). IHC showed diffuse strong cytoplasmic staining with pan-TRK. Staining for ALK, CD68 and CD163 were observed, whereas CD34, EMA, SMA, S100, and SOX10 were negative. Based on the unusual morphology for benign fibrous histiocytoma, the ALK expression by IHC and the fact that “epithelioid fibrous histiocytoma (EFH)” commonly demonstrate *ALK* fusions [41–43], NGS testing was performed and revealed *IRF2BP2*-*NTRK1* fusion (Fig. 3).

**Fig. 4:**
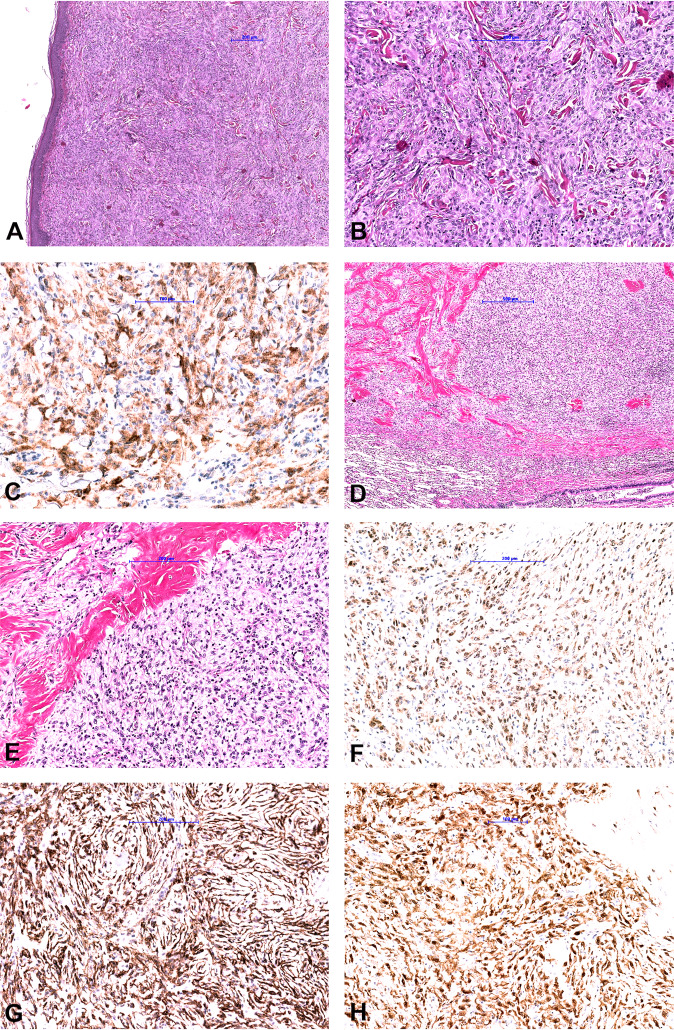
Morphological and immunohistochemical findings in cases 3 and 4. **a**, **b** Fibrohistiocytic skin tumor showed a monomorphic proliferation of spindle and epithelioid cells with mild nuclear atypia and eosinophilic cytoplasm with low mitotic activity**. c** Tumor cells showed diffuse strong cytoplasmatic pan-TRK staining. **d**, **e** Low-grade spindle cell lung sarcoma composed of monomorphic spindle cells arranged in a pattern-less pattern with prominent stromal and perivascular hyalinization. Tumor shows weak cytoplasmic pan-TRK staining (**f**) and diffuse strong co-expression of CD34 (**g**) and S100 (**h**).
4. The lung case presented as a low-grade spindle cell sarcoma composed of monomorphic spindle cells arranged in a pattern-less pattern, partly reminiscent of SFT with prominent stromal and perivascular hyalinization and co-expression of S100 and CD34 (Fig. 4d–h). However, IHC for STAT6 was negative. Staining with pan-TRK was diffuse weak cytoplasmic. In this case, an *RBPMS - NTRK3* fusion was found (Fig. 3).
5. High-grade spindle cell sarcoma was composed of cellular fascicles of atypical monotonous spindle cells having an MPNST-like appearance (Fig. 5). Mitoses were abundant (15/20HPF), and focal areas of necrosis were found. Immunohistochemically, tumor cells showed a strong diffuse cytoplasmic staining with pan-TRK. Focal staining with CD34 was observed, whereas the tumor was negative for S100 and SOX10. H3K27me expression was retained. Molecular analysis showed *TMB3-NTRK1* fusion (Fig. 3).

**Fig. 5:**
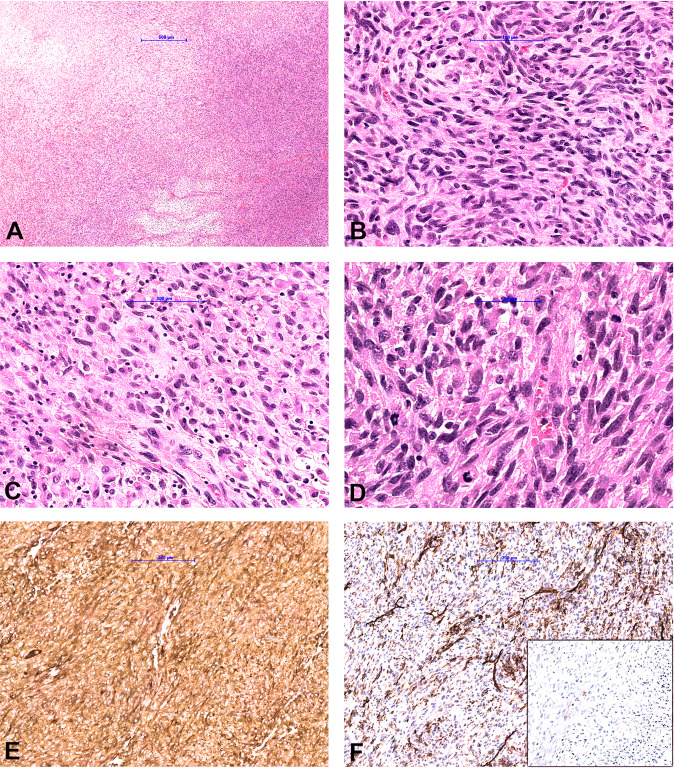
Morphological and immunohistochemical findings in case 5. **a** High-grade spindle cell sarcoma shows hypercellular and myxoid areas with focal necrosis. Tumor is composed of cellular fascicles of an atypical monotonous spindle (**b**) and epithelioid (**c**) cells with abundant mitotic activity (**d**). Immunohistochemically, tumor cells showed a strong diffuse cytoplasmic staining with pan-TRK (**e**), and focal staining with CD34 (**f**), while S100 was negative (**f** inset).

#### Treatment and follow-up

The patient with IFS received four months of chemotherapy (the combination of vincristine and actinomycin D) followed by a TRK inhibitor (Entrectinib) for over 12 months and has achieved a complete response with no sign of active disease on the last PET-CT and MRI scans at 16 months of follow-up. LFLNT, low-grade spindle cell lung sarcoma, and skin fibrohistiocytic lesion were resected in *toto* with negative margins. On their last follow-up, all patients were alive without recurrence after 20, 4, and 5 months, respectively. Lastly, the patient with high-grade sarcoma received total resection with adjuvant treatment, including irradiation and chemotherapy with Epirubicin/Ifosfamide and was free of the disease after 10 months follow-up.

## Discussion

A growing body of evidence recognizes the oncogenic role of chromosomal translocations involving *NTRK* genes found infrequently across a wide range of solid neoplasms. *NTRK*-rearranged mesenchymal neoplasms are an expanding group of tumors commonly arising in children or young adults. The tumors show morphologic heterogeneity and a range from benign to low and high-grade lesions [19–21]. They commonly recapitulate the morphology of infantile fibrosarcoma [8, 21, 39]. However, morphologies similar to inflammatory myofibroblastic tumor (IMT) [8, 39], solitary fibrous tumor (SFT) or MPNST can be seen [8, 39]. Recently, the locally aggressive LFLNT has been described, a tumor entity habouring *NTRK*-fusions, with similar morphology as dermatofibrosarcoma protuberans and co-expression of CD34, S100 and pan-TRK [8, 20, 39]. In this setting, a pan-TRK IHC can be used as a diagnostic tool. Of note, up to 90% of *NTRK*-rearranged mesenchymal tumors demonstrate more than one morphological pattern, a feature that can be a helpful clue in tumor recognition. In addition, most of the described tumors with *NTRK-*fusion show a consistent immunophenotype, namely: at least focal co-expression of S100, CD34, and pan-TRK, with negative SOX10 and H3K27me expression [19, 20, 29, 38, 39]. Tumors with overlapping morphology and IHC to *NTRK*-fusion tumors have been recently described to show *RET*- or *BRAF* fusions, also potential therapeutic targets [19, 44].

In our study, two cases (IFS and fibrohistiocytic dermal tumor) were S100 and CD34 negative. Two cases (LFLNT and low-grade spindle cell lung tumor) showed at least focal S100, and CD34 staining, and one case (high-grade spindle cell tumor) was only focally positive for CD34. In all cases, SOX10 and H3K27me were negative.

The principal mechanism of *NTRK1/2/3* gene fusions includes a fusion of the 3´ region of the *NTRK* gene containing the *NTRK* kinase domain with the critical tyrosine docking site with a 5´ fusion partner gene which is highly variable. *NTRK*- fusions lead to an active fusion protein that can be detected using IHC. Various molecular assays can detect *NTRK* rearrangements. As the detection of unknown and highly variable 5’ fusion partners is challenging, current recommendations suggest to use RNA-based NGS technologies, whereas RT-PCR or FISH should only be performed in tumors with a high frequency of recurrent *NTRK* fusions [37, 45]. By performing targeted RNA-sequencing approaches a spectrum of targetable oncogenic drivers can be simultaneously analyzed [6, 33, 37, 45, 46].

Unfortunately, due to expenses, NGS can not routinely be performed in every institution. Recently, it has been suggested that pan-TRK IHC should be used as a screening tool in institutions without available sequencing platform as an inexpensive, time- and tissue- efficient, surrogate method to screen for a potential *NTRK* gene fusion [37]. Positive IHC expression with a cut off of 1% should lead to an RNA-based NGS for detection/confirmation of the specific fusion in the appropriate clinical setting [30].

The monoclonal antibodies commercially available (none of them FDA approved) include rabbit TrkA (clone ab76291, Abcam), rabbit TrkB (clone J.977.7, Thermo Fisher and clone EPR17805-146, AbCam) and rabbit pan-TRK (clone EPR17341 from Abcam and Roche/Ventana). Even though the latter is available in two variants of the same clone, the pan-TRK antibody from Roche is, in contrast to Abcams´ antibody, a ready to use assay with fix procedure performed automatically. This should lead to results that are easy to reproduce and compare between different studies.

An overwhelming majority of studies used TrkA and pan-TRK (Abcam), the latter reacting with a C-terminus of TrkA, TrkB, and TrkC showing high specificity and sensitivity [6, 29, 30, 38, 39, 47]. Nevertheless, IHC evaluation may be challenging in tissue samples where TRK proteins are physiologically expressed, namely in neural and smooth muscle tissue. Moreover, as shown in the recently published studies, the staining patterns of the pan-TRK antibody (all studies used clone EPR17341 from Abcam) can vary in intensity and localization. Tumors harboring *NTRK1* rearrangements usually show strong, diffuse cytoplasmic staining. Nuclear staining (at least focal) has been described in tumors harboring *NTRK3* fusions and may be used as a surrogate of the presence of *NTRK3* fusions, however, the staining in these tumors can be weak [6, 29, 39]. Other patterns include cellular membrane staining, cytoplasmic staining, and nuclear expression with accentuation of the nuclear envelope [6, 29, 33, 39]. By comparing our TMA staining patterns with whole-tissue sections, a uniform staining pattern was observed. Recently, rare tumors with *NTRK* fusion were reported to lack TRK protein expression by IHC [29, 38]. By testing four of IHC negative cases where patients demographics might suggest a potential false-negative panTRK staining (younger age, spindle cell morphology) none of the cases showed an NTRK- fusion. Our study supports previous findings that false-negative cases seem to occur rarely [29, 38]. However, this cohort of nearly 500 soft tissue tumors has not entirely been screened by RNA-sequencing. Therefore, the sensitivity for this IHC assay cannot be accurately determined. False-positive staining with TRK antibodys can occur in non-*NTRK* fused tumors, mainly tumors with neural and smooth muscle differentiation, and some others namely: GIST, leiomyosarcoma, glioblastoma, neuroblastoma, a primitive myxoid mesenchymal tumor of infancy, synovial sarcoma, Ewing sarcoma, and fibrous hamartoma of infancy, SFT, soft tissue round cell sarcomas with *YWHAE* rearrangements, *BCOR* internal tandem duplications (ITD) and *BCOR-CCNB3* fusions and clear cell sarcomas of the kidney (another BCOR family tumor) as well as ossifying fibromyxoid tumors with *ZC3H7B-BCOR* fusion [29, 33, 38–40, 47].

**Fig. 6:**
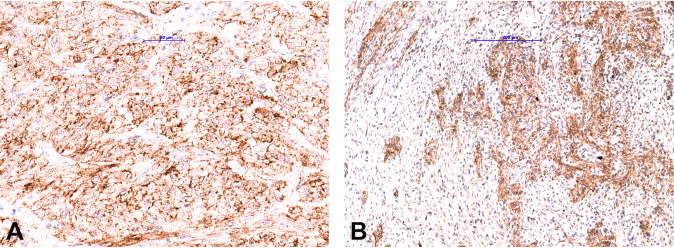
Pan-TRK immunohistochemical staining in gastrointestinal non-*NTRK* fusion tumors. Diffuse cytoplasmic/membranous pan-TRK expression in (**a**) gastrointestinal stromal tumor and (**b**) plexiform fibromyxoma of the stomach.

In our study, we are first to describe the use of a pan-TRK antibody (clone EPR17341, Assay, Roche, Ventana), an immunohistochemical assay performed automatically on Benchmark Ultra platform, in a large group of different soft tissue sarcomas and show similar results to previously published data using a different pan-TRK antibody (clone EPR17341, Abcam) [6, 29, 30, 38, 39]. In this study, weak focal cytoplasmic/membranous pan-TRK IHC staining occurd in extraskeletal myxoid chondrosarcomas, dedifferentiated liposarcoma, pleomorphic liposarcoma, and myxofibrosarcoma. In contrast, focal moderate to strong nuclear staining was found in synovial sarcoma and leiomyosarcoma. The latter also showed staining with dot-like aggregates. All these tumors underwent NGS, and none of them harbored an *NTRK* fusion. This result confirms data in the literature that *NTRK*-fusions, if present, are driver genomic alterations and that co-occurrence with additional driver genomic alteration is very limited [48]. To the best of our knowledge, *NTRK*-fusions in sarcomas with known genomic driver alterations e.g., in synovial sarcoma or extraskeletal myxoid chondrosarcomas are not reported. Moreover, we analyzed eight SFTs and none of them showed any pattern of staining. Of note, in our routine practice, we observed that GIST and plexiform fibromyxoma of the stomach can express diffuse moderate to strong cytoplasmic pan-TRK staining (Fig. 6) without demonstrating an *NTRK*-fusion by RNA sequencing.

To date, most of the fusions described in mesenchymal neoplasms involve *NTRK1* and *NTRK3* genes and include different fusion partner genes, most common being *ETV6*, *LMNA*, and *TPM3* [8, 29, 30, 36, 38, 49, 50]. In our cohort, two *NTRK3*-fusion-positive tumors are described: one with an *ETV6* fusion partner and one with *RBPMS*. The latter arose in the lung and showed only diffuse weak cytoplasmic pan-TRK staining, and an IHC pattern also demonstrated in mesenchymal tumors lacking *NTRK*-fusions. Nevertheless, the morphologic SFT-like pattern with the co-expression of CD34 and S100, as well as the lack of staining for STAT6 and SOX10, prompted us to perform RNA sequencing. The three *NTRK1-*fusion-positive tumors include *TMB3, LMNA*, and *IRF2BP2* as fusion partners. In all of these cases, diffuse strong cytoplasmic pan-TRK staining was observed.

Interestingly, we were able to detect an *NTRK*-fusion-positive skin lesion indicating that there is a spectrum of unusual benign histiocytic lesions in the skin demonstrating *NTRK-* fusions. We speculate that, similar to the “epithelioid fibrous histiocytoma” that harbors *ALK-*fusions in a high percentage of cases, there might also be a group of currently classified fibrohistiocytic lesions in the skin that will be shown to harbor *NTRK*-fusions. Further studies with a larger number of cases are needed to reveal whether a small subset of histiocytic/fibrohistiocytic lesions of the skin (e.g., EFH) [41–43] should be reclassified into mesenchymal tumors with *NTRK*-fusions.

The behavior of *NTRK*-rearranged tumors varies from benign to highly malignant. The benign neoplasm can be resected without further therapy, whereas malignant tumors may require treatment with TKI with an excellent response [12–18]. In our cohort, the patient with IFS was treated with TRK inhibitors and showed no sign of disease after 16 months. Patients with benign and low-grade tumors (#2–4) did not receive any further treatment after complete resection, and were free of disease after 20, 4, and 5 months. In the last case, after total resection of the high-grade sarcoma, the patient received adjuvant treatment (irradiation and chemotherapy) and was free of disease after 10 months.

In conclusion, *NTRK* rearranged mesenchymal neoplasms show variable morphologic patterns including LFLNT, SFT-like, IMT-like, IFS, DFSP-like, and MPNST like-spindle cell neoplasms and various histologic grades from low to high grade as well as benign forms. Primary *NTRK* -fusion tumors with mesenchymal origin can be seen also in the lung, and our data suggests that there might be a group of fibrohistiocytic tumors of the skin that might be placed in the group of *NTRK*-fusion tumors in the near future. The pan-TRK (clone EPR17341, RTU, Assay, Roche, Ventana) IHC is a reliable diagnostic marker that can be used as a surrogate marker for identification of *NTRK* fusion in the appropriate setting (young age, morphology) to help discover these rare tumors. Expensive RNA-based NGS for the detection/confirmation of specific fusions needs to be performed if patients are candidates for undergoing targeted therapy.

## References

[1] Amatu A, Sartore-Bianchi A, Bencardino K, Pizzutilo EG, Tosi F, Siena S (2019). Tropomyosin receptor kinase (TRK) biology and the role of NTRK gene fusions in cancer. Ann Oncol.

[2] Khotskaya YB, Holla VR, Farago AF, Mills Shaw KR, Meric-Bernstam F, Hong DS (2017). Targeting TRK family proteins in cancer. Pharm Ther.

[3] Farago AF, Taylor MS, Doebele RC, Zhu VW, Kummar S, Spira AI, et al. Clinicopathologic features of non-small-cell lung cancer harboring an NTRK gene fusion. JCO Precis Oncol. 2018. 10.1200/PO.18.00037. 10.1200/PO.18.00037 PMC6132056 30215037

[4] Knezevich SR, McFadden DE, Tao W, Lim JF, Sorensen PH (1998). A novel ETV6-NTRK3 gene fusion in congenital fibrosarcoma. Nat Genet.

[5] Knezevich SR, Garnett MJ, Pysher TJ, Beckwith, Grundy PE, Sorensen PH (1998). ETV6-NTRK3 gene fusions and trisomy 11 establish a histogenetic link between mesoblastic nephroma and congenital fibrosarcoma. Cancer Res.

[6] Solomon JP, Linkov I, Rosado A, Mullaney K, Rosen EY, Frosina D (2020). NTRK fusion detection across multiple assays and 33,997 cases: diagnostic implications and pitfalls. Mod Pathol.

[7] Bourgeois JM, Knezevich SR, Mathers JA, Sorensen PH (2000). Molecular detection of the ETV6-NTRK3 gene fusion differentiated congenital fibrosarcoma from other childhood spindle cell tumors. Am J Surg Pathol.

[8] Davis JL, Lockwood CM, Albert CM, Tsuchiya K, Hawkins DS, Rudzinski ER (2018). Infantile NTRK-associated mesenchymal tumors. Pediatr Dev Pathol.

[9] Church AJ, Calicchio ML, Nardi V, Skalova A, Pinto A, Dillon DA (2018). Recurrent EML4-NTRK3 fusions in infantile fibrosarcoma and congenital mesoblastic nephroma suggest a revised testing strategy. Mod Pathol.

[10] Tognon C, Knezevich SR, Huntsman D, Roskelley CD, Melnyk N, Mathers JA (2002). Expression of the ETV6 NTRK3 gene fusion as a primary event in human secretory breast carcinoma. Cancer Cell.

[11] Skalova A, Vanecek T, Sima R, Laco J, Weinreb I, Perez-Ordonez B (2010). Mammary analogue secretory carcinoma of salivary glands, containing the ETV6-NTRK3 fusion gene: a hitherto undescribed salivary gland tumor entity. Am J Surg Pathol.

[12] Drilon A, Laetsch TW, Kummar S, DuBois SG, Lassen UK, Demetri GD (2018). Efficacy of larotrectinib in TRK fusion-positive cancers in adults and children. N. Engl J Med.

[13] Drilon A, Ou SI, Cho BC, Kim DW, Lee J, Lin JJ (2018). Repotrectinib (TPX-0005) is a next generation ROS1/TRK/ALK inhibitor that potently inhibits ROS1/TRK/ ALK solvent-front mutations. Cancer Disco.

[14] Laetsch TW, DuBois SG, Mascarenhas L, Turpin B, Federman N, Albert CM (2018). Larotrectinib for paediatric solid tumours harbouring NTRK gene fusions: phase 1 results from a multicentre, open-label, phase 1/2 study. Lancet Oncol.

[15] Doebele RC, Drilon A, Paz-Ares L, Siena S, Shaw AT, Farago AF (2020). Entrectinib in patients with advanced or metastatic NTRK fusion-positive solid tumours: integrated analysis of three phase 1-2 trials. Lancet Oncol.

[16] Doebele RC, Davis LE, Vaishnavi A, Le AT, Estrada-Bernal A, Keysar S (2015). An oncogenic NTRK fusion in a patient with soft-tissue sarcoma with response to the tropomyosin-related kinase inhibitor LOXO-101. Cancer Disco.

[17] So YK, Chow C, To KF, Chan JKC, Cheuk W. Myxoid spindle cell sarcoma with LMNA-NTRK fusion: expanding the morphologic spectrum of NTRK-rearranged tumors. Int J Surg Pathol. 2020. 10.1177/1066896920905888. 10.1177/1066896920905888 32050835

[18] Hong DS, DuBois SG, Kummar S, Farago AF, Albert CM, Rohrberg KS (2020). Larotrectinib in patients with TRK fusion-positive solid tumours: a pooled analysis of three phase 1/2 clinical trials. Lancet Oncol.

[19] Suurmeijer AJH, Dickson BC, Swanson D, Zhang L, Sung YS, Cotzia P (2018). A novel group of spindle cell tumors defined by S100 and CD34 co-expression shows recurrent fusions involving RAF1, BRAF, and NTRK1/2 genes. Genes Chromosomes Cancer.

[20] Agaram NP, Zhang L, Sung YS, Chen CL, Chung CT, Antonescu CR (2016). Recurrent NTRK1 gene fusions define a novel subset of locally aggressive lipofibromatosis-like neural tumors. Am J Surg Pathol.

[21] Kao YC, Fletcher CDM, Alaggio R, Wexler L, Zhang L, Sung YS (2018). Recurrent BRAF gene fusions in a subset of pediatric spindle cell sarcomas: expanding the genetic spectrum of tumors with overlapping features with infantile fibrosarcoma. Am J Surg Pathol.

[22] Ricarte-Filho JC, Li S, Garcia-Rendueles ME, Montero-Conde C, Voza F, Knauf JA (2013). Identification of kinase fusion oncogenes in post-Chernobyl radiation-induced thyroid cancers. J Clin Invest.

[23] Cocco E, Benhamida J, Middha S, Zehir A, Mullaney K, Shia J (2019). Colorectal carcinomas containing hypermethylated MLH1 promoter and wild-type BRAF/KRAS are enriched for targetable kinase fusions. Cancer Res.

[24] Vaishnavi A, Capelletti M, Le AT, Kako S, Butaney M, Ercan D (2013). Oncogenic and drug-sensitive NTRK1 rearrangements in lung cancer. Nat Med.

[25] Wu G, Diaz AK, Paugh BS, Rankin SL, Ju B, Li Y (2014). The genomic landscape of diffuse intrinsic pontine glioma and pediatric non-brainstem high-grade glioma. Nat Genet.

[26] Milione M, Ardini E, Christiansen J, Valtorta E, Veronese S, Bosotti R (2017). Identification and characterization of a novel SCYL3-NTRK1 rearrangement in a colorectal cancer patient. Oncotarget.

[27] Pietrantonio F, Di Nicolantonio F, Schrock AB, Lee J, Tejpar S, Sartore-Bianchi A (2017). ALK, ROS1, and NTRK rearrangements in metastatic colorectal cancer. J Natl Cancer Inst.

[28] Prasad ML, Vyas M, Horne MJ, Virk RK, Morotti R, Liu Z (2016). NTRK fusion oncogenes in pediatric papillary thyroid carcinoma in northeast United States. Cancer.

[29] Hechtman JF, Benayed R, Hyman DM, Drilon A, Zehir A, Frosina D (2017). Pan-Trk immunohistochemistry is an efficient and reliable screen for the detection of NTRK fusions. Am J Surg Pathol.

[30] Gatalica Z, Xiu J, Swensen J, Vranic S (2019). Molecular characterization of cancers with NTRK gene fusions. Mod Pathol.

[31] Lezcano C, Shoushtari AN, Ariyan C, Hollmann TJ, Busam KJ (2018). Primary and metastatic melanoma with NTRK fusions. Am J Surg Pathol.

[32] Eguchi M, Eguchi-Ishimae M, Tojo A, Morishita TK, Suzuki K, Sato Y (1999). Fusion of ETV6 to neurotrophin-3 receptor TRKC in acute myeloid leukemia with t(12;15)(p13;q25). Blood.

[33] Chiang S, Cotzia P, Hyman DM, Drilon A, Tap WD, Zhang L (2018). NTRK fusions define a novel uterine sarcoma subtype with features of fibrosarcoma. Am J Surg Pathol.

[34] Brenca M, Rossi S, Polano M, Gasparotto D, Zanatta L, Racanelli D (2016). Transcriptome sequencing identifies ETV6-NTRK3 as a gene fusion involved in GIST. J Pathol.

[35] Wiesner T, He J, Yelensky R, Esteve-Puig R, Botton T, Yeh I (2014). Kinase fusions are frequent in Spitz tumours and spitzoid melanomas. Nat Commun.

[36] Amatu A, Sartore-Bianchi A, Siena S (2016). NTRK gene fusions as novel targets of cancer therapy across multiple tumour types. ESMO Open.

[37] Marchiò C, Scaltriti M, Ladanyi M, Iafrate AJ, Bibeau F, Dietel M (2019). ESMO recommendations on the standard methods to detect NTRK fusions in daily practice and clinical research. Ann Oncol.

[38] Rudzinski ER, Lockwood CM, Stohr BA, Vargas SO, Sheridan R, Black JO (2018). Pan-Trk immunohistochemistry identifies NTRK rearrangements in pediatric mesenchymal tumors. Am J Surg Pathol.

[39] Hung YP, Fletcher CDM, Hornick JL (2018). Evaluation of pan-TRK immunohistochemistry in infantile fibrosarcoma, lipofibromatosis-like neural tumour and histological mimics. Histopathology.

[40] Brodeur GM, Minturn JE, Ho R, Simpson AM, Iyer R, Varela CR (2009). Trk receptor expression and inhibition in neuroblastomas. Clin Cancer Res.

[41] Walther C, Hofvander J, Nilsson J, Magnusson L, Domanski HA, Gisselsson D (2015). Gene fusion detection in formalin-fixed paraffin-embedded benign fibrous histiocytomas using fluorescence in situ hybridization and RNA sequencing. Lab Invest.

[42] Dickson BC, Swanson D, Charames GS, Fletcher CDM, Hornick JL (2018). Epithelioid fibrous histiocytoma: molecular characterization of ALK fusion partners in 23 cases. Mod Pathol.

[43] Creytens D, Ferdinande L, Van Dorpe J (2017). ALK rearrangement and overexpression in an unusual cutaneous epithelioid tumor with a peculiar whorled “perineurioma-like” growth pattern: epithelioid fibrous histiocytoma. Appl Immunohistochem Mol Morphol.

[44] Antonescu CR, Dickson BC, Swanson D, Zhang L, Sung YS, Kao YC (2019). Spindle cell tumors with RET gene fusions exhibit a morphologic spectrum akin to tumors with NTRK gene fusions. Am J Surg Pathol.

[45] Penault-Llorca F, Rudzinski ER, Sepulveda AR (2019). Testing algorithm for identification of patients with TRK fusion cancer. J Clin Pathol.

[46] Albert CM, Davis JL, Federman N, Casanova M, Laersch TW (2019). TRK fusion cancers in children: a clinical review and recommendations for screening. J Clin Oncol.

[47] Kao YC, Sung YS, Argani P, Swanson D, Alaggio R, Tap W, et al. NTRK3 overexpression in undifferentiated sarcomas with YWHAE and BCOR genetic alterations. Mod Pathol. 2020. 10.1038/s41379-020-0495-2. 10.1038/s41379-020-0495-2 PMC7329614 32034283

[48] Jiao X, Lokker A, Snider J, Castellanos E, Nanda S, Fisher V (2019). Co-occurrence of NTRK fusions with other genomic biomarkers in cancer patients. Ann Oncol.

[49] Cocco E, Scaltriti M, Drilon A (2018). NTRK fusion-positive cancers and TRK inhibitor therapy. Nat Rev Clin Oncol.

[50] Pavlick D, Schrock AB, Malicki D, Stephens PJ, Kuo DJ, Ahn H (2017). Identification of NTRK fusions in pediatric mesenchymal tumors. Pediatr Blood Cancer.

